# Circulating miRNAs as potential liquid biomarkers for pediatric gliomas

**DOI:** 10.1038/s41390-025-04320-6

**Published:** 2025-08-20

**Authors:** Dikla Rogachevsky, Michal Yalon, Amos Toren, Ruty Mehrian-Shai

**Affiliations:** 1 Faculty of Medical and Health Sciences, Tel-Aviv University, Tel Aviv, Israel; 2 Pediatric Hemato-Oncology, Sheba Medical Center, Tel Ha’shomer, Israel

## Abstract

**Background:**

Current diagnostic and monitoring of pediatric brain tumors rely on invasive tissue biopsies and imaging, highlighting the need for non-invasive alternatives. For that matter, liquid biopsy is a promising method. This pilot study investigated the potential of circulating miRNAs to serve as non-invasive biomarkers of pediatric glioma tumors.

**Methods:**

The levels of mir-182-5p, mir-10b-5p, mir-106b-3p, mir-25-3p, and mir-21-5p were quantified in plasma samples from 68 pediatric gliomas patients and 12 Healthy controls using Real-time PCR. The results were assessed by differential and ROC curve analysis. Additionally, the involvement of mir-182-5p in pediatric high-grade gliomas aggressiveness was examined by functional studies using two cellular models.

**Results:**

All five circulating miRNAs demonstrated increased levels in pediatric glioma patients compared to HCs, significant correlation with Gliomas, and differential expression between various glioma subtypes. These miRNAs presented potential diagnostic discrimination between HCs and pediatric gliomas, as well as between different glioma subgroups. mir-182-5p showed the highest correlation to glioma and induction of migration as well as invasion ability of pediatric diffuse high-grade glioma 4 (*pHGG-4)* cells in vitro.

**Conclusions:**

Our findings present circulating miRNAs as promising biomarkers and treatment targets that, with further research, may be clinically utilized for monitoring pediatric glioma tumors.

**Impact:**

- This study demonstrates the potential of circulating miRNAs as valuable biomarkers for pediatric glioma detection and monitoring.
- Five examined circulating miRNAs were differentially expressed in pediatric glioma patients compared to controls.
- These miRNAs were correlated with glioma and could distinguish healthy from gliomas patients.
- Statistical analysis suggested these miRNAs may also distinguish between various glioma subtypes.
- Inhibition of the most promising circulating miRNA, miR-182-5p, decreased migration ability and invasiveness of pediatric high-grade glioma IV cells.
- In the future, the miRNAs studied could have applications as biomarkers for clinical management and treatment targets.

## Introduction

Central nervous system (CNS) tumors, are the most common form of childhood (0–14 years) and adolescent (15–19 years) solid cancers.^1–3^ Among these, pediatric gliomas are the most prevalent, accounting for 31.1–51.6% of CNS tumors.^4^ These tumors present a broad spectrum of clinical courses, from slow-growing tumors to highly aggressive malignancies with the highest mortality rate.^1,2,4,5^ Glioma classification primarily relies on histological grading, which categorizes these tumors into two major groups: low-grade gliomas (LGGs) and diffuse high-grade gliomas (HGGs). While advancements in surgery and adjuvant therapy have yielded improved 5-year survival rates that exceed 75% for pediatric LGGs, the prognosis for pediatric-HGGs (pHGGs) remains dismal (around 10%).^1,6^

In most cancers, early detection of a tumor or its recurrence, as well as accurate assessment of the tumor response to therapy, can be important for improving disease outcome.^7,8^ To date, the main clinical diagnostic modality for pediatric brain tumors is imaging by computed tomography (CT) or magnetic resonance imaging (MRI), which is finalized by histopathologic and molecular analyses of a tumor sample, received from resection surgery or biopsy.^9^ Yet, tumor resection and biopsies are invasive neurosurgical procedures, and in many pediatric glioma cases, especially in brainstem-LGG, diffuse intrinsic pontine gliomas (DIPGs), and optic pathway, even stereotactic biopsy is complicated, due to tumor location.^6,10,11^ Additionally, biopsies only capture a small portion of the tumor, potentially missing genetically diverse sub-clones, and even surgically-excised tumors are restricted to limited numbers of representative slices, hence do not fully capture the intra-tumoral heterogeneity.^10,12^ Given these traditional diagnostic methods limitations, there is a pressing need for a non-invasive method that can facilitate early detection, measure disease burden, and monitor the treatment response of pediatric gliomas. Liquid biopsy, which involves the analysis of biomarkers found in blood, CSF, and other bodily fluids, offers a promising non-invasive alternative.^13^

Several informative molecular markers are currently considered as potential biomarkers for liquid biopsy, including circulating tumor cells, circulating tumor DNA (ctDNA), tumor-derived extracellular vesicles (EVs), and circulating miRNAs.^13,14^ To date, the most studied liquid biomarker for pediatric CNS tumors is ctDNA detected in cerebrospinal fluid (CSF).^11,13^ ctDNA consists of cell-free DNA fragments released from tumor cells through apoptosis, necrosis, and budding, and it has been used by researchers to detect tumor-associated copy-number variations, tumor-specific driver mutations, and epigenetic alterations. CSF ctDNA has been shown to positively correlate with the presence of active disease and disease burden, and current studies have established it as a highly effective biomarker for adult and pediatric CNS tumors, including glioblastoma (GB), medulloblastoma, and diffuse midline/brainstem gliomas.^11,15–17^

Using CSF ctDNA as a liquid biomarker may be feasible and beneficial, however, CSF collection is logistically complex, as it requires pediatric patients to undergo serial lumbar puncture, a procedure that carries a relatively high risk.^11^

Studies comparing the efficacy of ctDNA detection in CSF with that in plasma revealed that using plasma ctDNA as a biomarker for CNS tumors is inferior to CSF ctDNA. The CSF is a richer source of ctDNA, due to CNS tumors proximity, and due to the blood-brain barrier (BBB). The BBB acts as a physiological shield that significantly restricts ctDNA passage from the CNS tumor microenvironment into the bloodstream. As a result, plasma ctDNA has limited sensitivity for detecting and monitoring CNS malignancies compared to CSF ctDNA, which is in direct contact with the tumor microenvironment.^11,15,18^ The complexities of ctDNA collection encourage further search for easily detectable plasma biomarkers that would optimize pediatric glioma diagnosis and potentially replace current monitoring methods. Obtaining plasma samples is significantly less invasive, hence more feasible option for regular monitoring in clinical settings.

MicroRNAs (miRNAs), short (~22 nucleotides) single-stranded non-coding RNAs, are currently considered as promising plasma (circulating) biomarkers.^19–25^ miRNAs are secreted by most cell types, including by tumor cells, into the extracellular fluids, and can be transported through circulation to remote target cells, functioning as chemical messengers that mediate cell-cell communication.^20,26^ In target cells, they regulate numerous biological processes through their post-transcriptional regulatory function.^8,26^ The circulating miRNAs can be detected in biological fluids such as plasma, serum, and CSF, existing in remarkably stable state inside EVs, small membrane vesicles such as exosomes, in apoptotic bodies, or in complex with specific proteins.^8,20,26,27^

Circulating miRNAs exhibit aberrant expression patterns under pathological conditions, such as cancer and other diseases, and each disease or tumor type demonstrates specific plasma miRNA fingerprints. These findings indicate that differentially expressed circulating miRNAs can serve as biomarkers for cancer diagnosis.^8,19,21,23,26,28,29^

Recent studies have identified differentially expressed miRNAs released into bodily fluids by cells of CNS tumors, such as adult GBs, medulloblastoma, pediatric LGGs, and DIPGs, while encapsulated within exosomes that facilitated their passage even across an intact BBB into the circulation.^11,19^ These circulating miRNAs have been demonstrated as a promising diagnostic tool for adult GBs and pediatric LGGs, and as a prognostic tool for predicting tumor status and progression for pediatric DIPG.^12,30,31^ The advantages of their differential expression patterns, their ability to cross an intact BBB (unlike ctDNA), and their stability in bodily fluids highlight plasma miRNAs as potentially valuable biomarkers for the diagnostic detection of pediatric gliomas.

In this pilot study, we analyze plasma samples from both healthy children and pediatric glioma patients of varying grades for circulating miRNAs. Our study focuses on miR-182, miR-10b-5p, miR-182-5p, mir-21-5p, mir106-3p, and mir-25-3p. These five miRNAs were identified by Jha et al.^32^ as being among the 20 most upregulated miRNAs in pHGG-4 snap-frozen tumor samples.^32^ Later, Ebrahimkhani et al.^19^ have reported elevated plasma levels of these miRNAs, except miR-21-5p, in adult GB patients serum specimens, collected preoperatively from patients with histologically confirmed glioma tumors.^19^

Lastly, in our study, we also explored the potential involvement of miR-182-5p in pHGG-4 aggressiveness.

## Materials and methods

### Liquid biopsy samples

Blood samples were obtained from pediatric brain tumor patients and healthy control (HC) at Sheba Medical Center. Patients’ samples were collected from the Hemato-Oncology Department and clinic, while HC samples (children, adolescents, and young adults, without tumors or endocrine abnormalities) were obtained from the International Congenital Heart Center, the Pediatric Endocrinology Department, and the Sheba Blood Bank. The HC group included children without malignancies and with well-managed health conditions. All blood samples were donated with consent, with Helsinki approval 7259-09-SMC, 7079-09-SMC, 4560-17-SMC. The consent forms for underage were signed by parents or legal guardians of the participants. The overall study protocol was reviewed and approved by the Institutional Review Board to ensure compliance with ethical guidelines.

The research cohort consisted of 80 pediatric plasma samples, including 12 pediatric HCs and 68 pediatric glioma patients, of which 40 LGGs, 12 LGGs located in the brain stem or spinal cord (BS&SC.LGGs), and 17 patients with *pHGG*. Each blood sample was collected in an EDTA-containing blood collection tube. The received samples were processed within 2 h of blood collection for obtaining the plasma, as described in the plasma separation protocol. The obtained plasma samples were used for detecting selected miRNAs’ expression.

### Plasma separation and storage

The plasma was separated from the blood sample by using Lymphoprep (Serumwerk Berburg AG, Alere Technologies AS, Oslo, Norway) and Leucosep 12 ml sterile tube. The tubes were centrifuged in 1000 × *g* for 1 min at room temperature, to spin down the Lymphoprep. Each blood sample was passed gently to a prepared Leucosep tube and then centrifuged for 10 min at 2400 rpm (brake off) at room temperature. The upper plasma phase was then carefully transferred in aliquots of 200 μl to freezer tubes and stored in a −80 °C freezer.

### Purification of total RNA from plasma

Total RNA, including miRNAs, was extracted from the plasma samples using miRNeasy Serum/Plasma Advanced Kit (QIAGEN). Briefly, the plasma samples were thawed on ice and the RNA extraction was performed according to the manufacturer’s protocol, to receive 20 μl of Total Plasma RNA in ultrapure double-distilled H_2_O.

### Relative evaluation of specific plasma miRNAs

The expression of specific miRNAs in plasma samples was evaluated using the miRCURY LNA miRNA SYBR® Green PCR System (QIAGEN), 5 µl plasma RNA was used for universal reverse transcription (RT) reaction followed by amplifying the specific miRNA of interest, in a real-times PCR (qPCR) assay using forward and reverse LNA enhanced miRNA specific qPCR primers (miRCURY LNA PCR primer assay, ordered from QIAGEN; Catalog no. 339306) which is uniquely designed for plasma miRNA profiling. The RT reaction samples are prepared with the addition of a spike-in RNA (UniSp6 from the kit), which is used as a reference for normalization. The spike-in UniSp6 reference is detected using UniSp6 RNA spike-in Control Assay. The reaction for every control/experimental cDNA sample was performed in triplicate.

### Cell lines

In our research, we used two well-characterized cell line models, originally established from two molecularly different *pHGG-4* patients, the SF188 and KNS42 cell lines.^33^ The pediatric SF188 cell line was kindly gifted by Cynthia Cowdrey, University of California, San Francisco, and was cultured in DMEM medium (Gibco) supplemented with 10% FBS and 1% Pen-strep. The *pHGG-4* cell line, KNS42, was obtained from the JCRB (Japan Cancer Research Resources) cell bank. The cells were cultured in MEME medium (Biological Industries) supplemented with 5% FBS, cultured in DMEM medium (Gibco) supplemented with 5% FBS (Gibco) and 1% penicillin–streptomycin (Pen-Srep), and 1% L-Glutamine. Normal Human astrocytes (NHA) (CC-2565, Lonza) were purchased from Lunza. These cells were grown, according to Lunza protocols, in Clonetics Astrocyte Cell System (Lonza), which is Astrocyte Medium BulletKit—(Clonetics AGM BulletKit (CC-3186)).

### Inhibition of miRNA’s expression

The mir182 inhibition experiments were done using the miRCURY LNA™ microRNA Inhibitor specific for mir-182-5p (EXIQON) (mir182 inhibitor), and a scrambled oligonucleotide that is used as Negative control (Negative control inhibitor A, EXIQON). Briefly, SF188 or KNS42 cells were seeded in 12-well plates and incubated in 37 °C, while transfection liquid was prepared. Transfection was performed using aliquot of transfection liquid prepared from 50 nM Negative Control or mir-182 inhibitor, and HiPerFect transfection reagent dilute in the appropriate serum free medium according to the manufacturer’s protocol. The SF188 and KNS42 plates were then incubated in 37 °C for 48 h and 72 h, respectively. In each experiment, every transfection assay was done with 6–12 repeats for the negative control or mir182 inhibitor. When harvested, every two wells were united to one technical sample.

### Trans-well migration and invasion assays

Cell samples were harvested from transfection assays and counted. Each cell sample, 8 × 10^5^ SF188 cells or 5 × 10^5^ KNS42 cells (in triplicates), was suspended in 200 μl serum free medium, and seeded in a 24-well insert (Falcon cell culture 8μm inserts, corning). The insert was then put in 750 μl full supplemented medium. After 24 h incubation in 37 °C, 5% CO_2_, the inserts were fixed and dyed in Giemsa. Images were taken from five different areas of each insert/treatment using EVOS microscopy (×10), and cell were counted using ImageJ program. The results are the average of experiment repeats that were normalized to the control.

#### Trans-well migration assay

Before seeding KNS42 cell, each insert was filled with 100 μl serum-free medium and then with 200 μl of the right cell suspension.

#### Trans-well invasion assay

A day before the assay performance, insert membrane was coated with 100 μl extracellular matrix made from BME (Cultrex) diluted (3 mg/ml for KNS42 and 1 mg/ml for SF188 cells) in serum free medium and incubated overnight in 37 °C, 5% CO_2_ in 24 wells plate. The next day the assay proceeded as described.

### Wound healing scratch assay

This assay was performed for SF188. Briefly, Wells (12 well plates) seeded with control or mir-182-5p inhibited SF188 cells, were scratched using a sterile 1000 μl tip to create a dimeter long scratch. Images of the scratches were taken at 0, 21 and 26 h time points, using EVOS microscope (×4). Migration rate was calculated as the percent gap-change between 0 and 26 h gap, using ImageJ program.

### Statistical analysis

Statistical analysis and graphs/plots were generated with GraphPad Prism software or Excel. Data are presented as mean with standard error for all measurements. Statistical significance was determined by one-tailed unpaired Student’s *t* test (for two groups) or one-way ANOVA with Tukey’s multiple comparison test, with *p* < 0.05 considered statistically significant.

Receiver operating characteristic (ROC) curve analysis was used to evaluate the diagnostic ability of each of the examined circulating miRNA. Spearman’s correlation coefficient was used to determine the association between each miRNA and glioma. Pairwise correlations were used to determine the correlation between the five miRNAs.

## Results

### Circulating miRNAs in pediatric Glioma patients

In this study, we investigate the expression of miR-10b-5p, miR-182-5p, miR-21-5p, miR-106-3p, and mir-25-3p as potential blood-based biomarkers for pediatric glioma. Our study cohort includes 80 pediatric subjects with brain tumors, of whom 68 were diagnosed with gliomas, and 12 were pediatric HCs. The case cohort demographic and clinical data summary is presented in Supplementary Table (ST1). The plasma is isolated from whole blood and the specific miRNA relative levels are quantified.

**Fig. 1:**
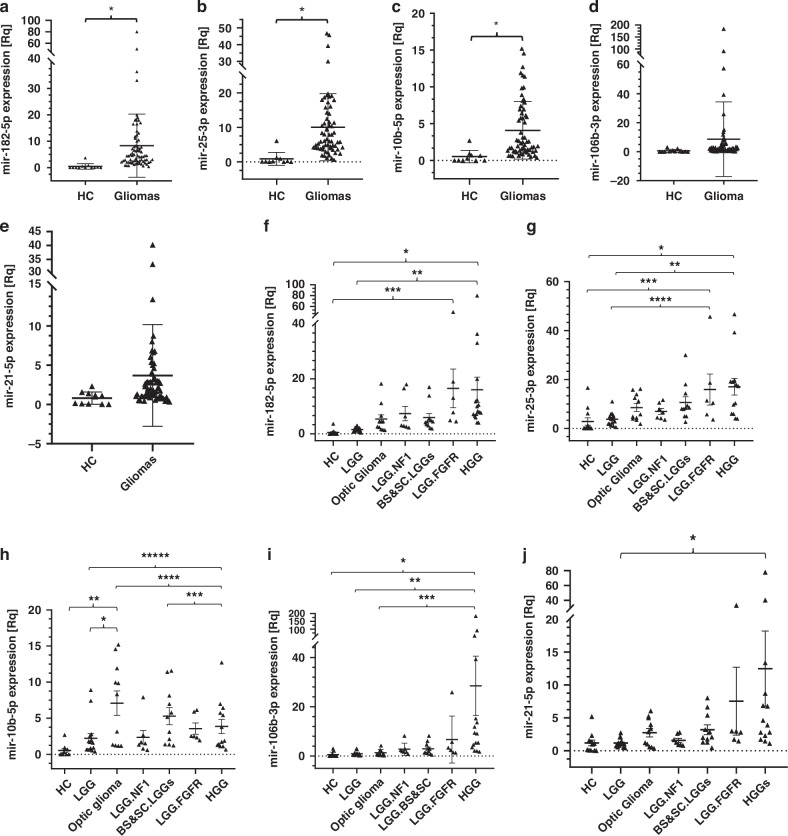
Relative expression of circulating miRNAs in pediatric gliomas. The relative plasma expression levels of mir-182-5p, mir-25-3p, mir-10b-5p, mir-106b-3p, and mir-21-5p, are presented for Healthy control individuals and Glioma patients. **a** (*p* < 0.01); **b** (*p* < 0.001); **c** (*p* < 0.005); **d**, **e** present HCs compared to Total glioma patients (statistical analysis with *t* test), while in (**f**–**j**) present the circulating expression of each miRNA is presented separately, for the different pediatric glioma group: pediatric LGG, Optic Glioma, LGG + NF1 germline mutations (LGG.NF1), LGG + FGFR Aberrations (LGG.FGFR), Brain Stem and spinal cord LGGs (BS&SC. LGGs), and for HGGs Brain Stem and pHGG-IV (HGG). The results are presented in Relative quantities (Rq = 2^−ΔΔCt^).

A comparative analysis of total glioma patients and HCs reveals a significant differential expression pattern for all five circulating miRNAs. Specifically, pediatric glioma patients exhibit significantly elevated levels of 182-5p (*p* < 0.05), mir-25-3p (*p* < 0.005), and mir-10b-5p (*p* < 0.005) compared to HCs (Fig. 1a–c). Although circulating mir-106b-3p and mir-21-5p are also elevated in pediatric glioma patients (Fig. 1d, e), the overall mean expression levels of these miRNAs in the entire glioma patient cohort do not significantly differ from those of HCs. Comprehensive results analysis reveals heterogeneous miRNAs expression in the LGG patients’ group. While most LGG patients exhibit low plasma levels of all five miRNAs, a subset of the LGG group displays notably elevated miRNA expression. Molecularly, LGGs most commonly exhibit BRAF translocations or fusions, and to lesser extent *BRAF* activating mutations. Other alterations include *NF1* mutations and the *FGFR1* aberrations which represent LGGs with poor prognosis.^6,34,35^ Consequently, we further stratify the LGG group into distinct subgroups, based on the tumor site or molecular characteristics: LGG, LGG with NF1 (LGG.NF1), optic gliomas (OG), brain stem and spinal cord LGGs (BS&SC.LGGs), and LGG with FGFR aberrations (LGG.FGFR). Circulating miRNA expression for each pediatric group are summarized in Fig. 1f–j and the plasma expression averages, errors, and ANOVA results for each circulating miRNA in Supplementary Table (ST2A–E).

One-way ANOVA analysis of the circulating miRNA expression reveals significantly higher mean levels of miR-182-5p in LGG.FGFRs (Rq =1 5.927) compared to HCs (Rq = 0.468), and in HGGs (Rq = 16.034) compared to LGG (Rq = 1.552) or HCs. The mir-25-3p mean level is significantly elevated in HGGs (Rq = 17.09) and LGG.FGFRs (Rq = 15.927) compared to HCs (Rq = 0.889) or LGGs (Rq = 3.32). The mean mir-10b-5p level was significantly higher in OGs (Rq = 7.076) compared to HCs (Rq = 0.526), LGGs (Rq = 2.127), or HGGs (Rq = 3.789). Additionally, the BS&SC.LGGs exhibit significantly higher mean mir-10b-5p level (Rq = 5.288) compared to HC or HGGs, while HGGs display significantly higher mean level compared to LGGs. The mean mir-106b-3p level in HGGs (Rq = 28.5) is significantly higher compared to HCs (Rq = 0.5602), LGGs (Rq = 0.924), or OGs (Rq = 1.331). While not statistically significant compared to HCs, the mean miR-21-5p level in HGG patients (Rq = 12.48) is ~3.9-fold higher than that of BS&SC.LGG patients (Rq = 3.19) and 4-6-fold higher than other glioma patient groups. ANOVA analyses reveal significant differences only between LGG and HGGs.

### The potential of circulating miRNAs to discriminate glioma from HCs

**Fig. 2:**
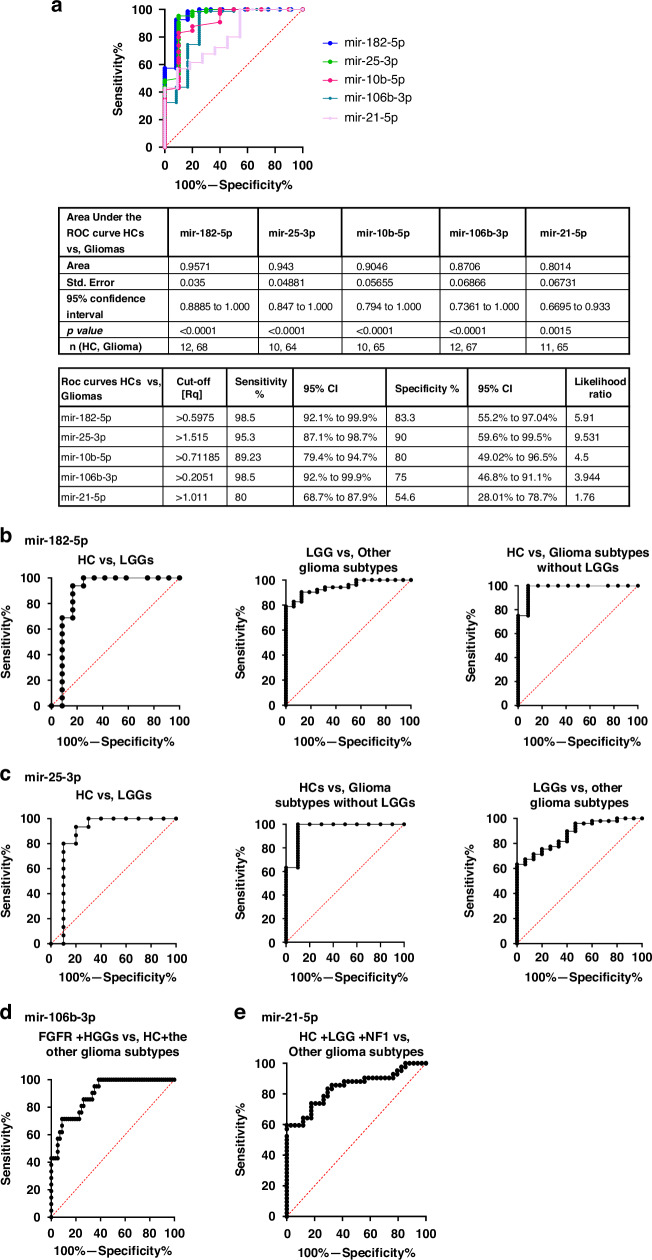
Receiver operator characteristic (ROC) curves. For each miRNA to evaluate their potential as liquid biomarkers for pediatric glioma patients’ diagnostic discrimination. **a** ROC curves analysis demonstrating the power of each miRNA to discriminate Gliomas from healthy controls; **b** ROC curves analysis for circulating mir-182-5p demonstrating diagnostic discrimination between pediatric HCs, pediatric LGGs, and a third pediatric group that includes OG, LGG.NF1 (NF1), LGG.FGFRs (FGFR), BS&SC.LGG (BS&SC) and HGGs; **c** ROC curves analysis for circulating mir-25-3p, presenting diagnostic discrimination between pediatric HCs, LGGs and a third group of pediatric gliomas that includes OG, NF1, FGFRs, BS&SC and HGGs; **d** ROC curves analysis for circulating mir-106b-3p, presenting potential diagnostic discrimination between two statistically different pediatric groups, one comprise of FGFR and HGGs, and the other include HC with the other glioma subtypes (HC + LGGs + OGs + NF1 + BS&SC); **e** ROC curves analysis for circulating mir-21-5p present potential discrimination between two statistically different pediatric groups, the first includes HCs, LGG and NF1 and the second comprise of the other glioma subtypes (OG + FGFRs + BS&SC + HGGs).

ROC curves are generated for each miRNA to evaluate their diagnostic ability to distinguish pediatric glioma patients from HCs. The ROC curve analyses compare the relative quantities (Rqs) of each miRNA in all the glioma patients to those in healthy individuals. The results (Fig. 2a) demonstrate excellent discriminatory power for mir-182-5p (Area under curve (AUC) = 0.9571), mir-25-3p (AUC = 0.943), and mir-10b-3p (AUC = 0.9046).

Although according to *t* test results, the difference between HCs and total glioma groups in mir106b-3p and mir-21-5p is not statistically significant, the ROC curves demonstrate highly good (0.8706; *p* < 0.0001) and good (0.8014; *p* < 0.0015) discriminator values, respectively. Overall, these results present a strong ability for mir-182-5p, mir-25-3p, and mir-10b-3p to diagnostically discriminate between Healthy individuals and pediatric glioma patients.

### The miRNAs’ ability to discriminate between the different pediatric glioma groups and HC

The capability to discriminate between the different pediatric groups, including HCs, is evaluated for each of the five circulating miRNAs, using the ROC curves analysis.

mir-182-5p analyses (Fig. 2b) reveal diagnostic discrimination between HCs, LGGs and a third group that includes all other glioma subtypes. mir-182-5p distinguished HCs from LGGs with highly good discriminatory value (AUC = 0.8854), from the other glioma subtypes with an excellent clinical value (0.979167), and last, it distinguishes LGGs from the other glioma subtype group with an excellent clinical diagnostic value (0.946) (Fig. 2b). Analyses between HCs and each of the glioma subtypes demonstrate highly good to excellent discriminatory values (AUC between 0.8854 and 1); however, HGGs present no discriminatory power with LGG.FGFR (AUC = 0.5196), and no useful discriminatory value with LGG.NF1 (Supplementary Fig. SF1A).

mir-25-3p analyses present diagnostic discrimination between pediatric HCs, LGGs, and a group comprising of all other glioma subtypes (Fig. 2c). It distinguishes, with highly good clinical values, between HCs and LGGs (AUC = 0.873) and between LGGs and the other glioma subtypes group (0.875), and with an excellent discriminatory value, between HCs and the other glioma subtypes group (0.963). Analyses comparing between HC and glioma subtypes present limited discrimination abilities (Supplementary Fig. SF1B), with low to no clinical values for comparing HGGs with other glioma subtypes (except LGGs).

mir-10b-5p analyses demonstrate diagnostic discrimination, with an excellent discriminatory value, only between pediatric HCs and the total pediatric glioma patients, but present limited ability to distinguish between different glioma subtypes (Fig. 2d). It discriminates HCs from different glioma subtypes with good to excellent discriminatory values, and LGGs with most glioma subtypes with moderate discriminatory values, except for LGG.NF1, which has no clinical value (Supplementary Fig. SF1C). However, HGGs present no clinical value with glioma subtypes except LGGs.

miR-106b-3p analyses present potential diagnostic discrimination, with highly strong diagnostic value (0.8947), between two statistically different pediatric groups, one consisting of HCs, LGGs, and LGG.NF1, and BS&SC. LGGs, and the other of LGG.FGFR and HGGs (Fig. 2d). This observed discrimination is attributed to the limited diagnostic capability of miR-106b-3p to differentiate between HCs and glioma subtypes (Supplementary Fig. SF1D), in this pilot.

The analyses for mir-21-5p present limited abilities to discriminate between HCs and different glioma subtypes (Supplementary Fig. SF1E) in this pilot study. Instead, the analyses suggest potential discrimination between two statistically different pediatric groups, one that included HCs, LGG and LGG.NF1 and a second that comprises of all other glioma subtypes (Fig. 2e).

The analyses conducted for the five circulating miRNAs reveal their potential as indicators for pediatric gliomas. While the number of healthy individuals and patients in each glioma group is quite small, the results, which are summarized in Table 1 are encouraging. These results can serve as a starting point for developing an algorithm that utilizes the five circulating miRNAs as biomarkers for diagnosing or monitoring pediatric gliomas. The initial step of a potential algorithm is to signal the possible presence of glioma tumor, using the relative expression of circulating mir-182-5p, mir-25-3p, and mir-10b-5p, which present excellent diagnostic discriminatory power compared to healthy pediatric individuals. Relative expression values above the cutoff Ct value of mir-182-5p, mir-25-3p, and mir-10b-5p (suggested here as >0.5975, >1.5150, and >0.71185, respectively) will raise a reasonable suspicion of a glioma tumor presence.

**Table 1:** Summary of receiver operating characteristic (ROC) curve analysis results, suggesting diagnostic performance for the selected microRNAs.

The table presents cutoff values in qPCR relative quantities (Rq, 2^−^^ΔΔCt^), sensitivity (%), and specificity (%) for each marker, according to the pilot results.

The following potential steps may further discriminate glioma groups. These steps rely on the relative expression of circulating mir-182-5p and mir-25-3p, both of which present similar and highly good to excellent diagnostic discrimination patterns, distinguishing between HCs, LGG, and other glioma subtypes. The other two circulating miRNAs, mir-106b-3p and mir-21-5p, present lower capability to discriminate pediatric glioma patients from HCs. However, according to the pilot results, these two miRNAs may increase the suspicion of glioma, hence can be used as diagnostic support.

### mir182-5p inhibition decreases migration ability and invasiveness of pHGG-IV tumor cells

**Fig. 3:**
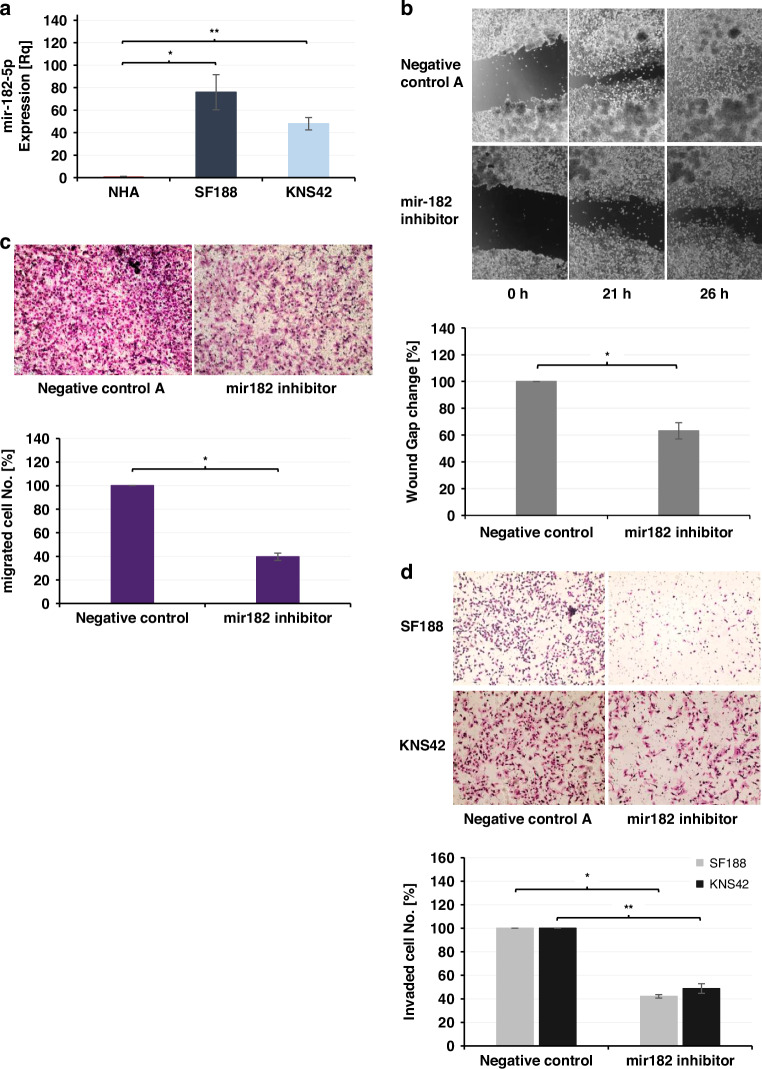
mir182 in pHGG IV GB cell lines. **a** mir182 expression in SF188 and KNS42 cells (*p < 0.0005; **p < 0.00001); **b** scratch wound healing assay showing mir182 inhibition (*p < 0.05) decreased migration ability of SF188 cells. **c** Trans-well migration assay demonstrating mir182 inhibition decreased KNS42 cells’ migration ability (*p < 0.0005); **d** Trans-well Invasion assays revealing that mir182 inhibition decreased the invasiveness of SF188 and KNS42 cells (*p < 0.05; **p < 0.005).

Our results indicated that mir-182-5p has the greatest potential as a biomarker for pediatric gliomas. It is highly expressed in the plasma of pediatric glioma patients compared to HCs, with significantly higher expression in HGGs compared to LGGs. Moreover, mir-182-5p demonstrates the strongest correlation with glioma among the five miRNAs studied. miR-182-5p is also one of the most aberrantly overexpressed miRNAs in *pHGG-4* tumors,^32^ however, its role in pediatric gliomas is yet unpublished. To explore the potential role of miR-182-5p in regulating *pHGG-4* aggressiveness, we first examine its expression in two *pHGG-4* models, the SF188 and KNS42 cells. The results show that mir-182-5p expression is significantly upregulated in both cell lines, compared to NHA cells (Fig. 3a). To evaluate the functional significance of miR-182-5p in *pHGG-4* aggressiveness, we decrease mir182 expression by transfecting SF188 and KNS42 cells with hsa-miR-182-5p antisense LNA oligonucleotides and examine their survival, migration ability, and invasiveness. Our results demonstrate that mir-182-5p inhibition does not impact the survival of SF188 or KNS42 cells (Supplementary Fig. SF2), despite previous reports showing that mir-182-5p promotes proliferation in melanoma, breast, and prostate cancers.^36–38^ The migration capability of mir-182-5p inhibited SF188 and KNS42 cells is assessed using scratch wound healing or trans-well migration assays, respectively. The results demonstrate that mir-182-5p inhibition reduce the migration ability of both SF188 cells, by 37%, and KNS42 cells, by 60% (Fig. 3b, c). To evaluate the effect of mir-182-5p on *pHGG-4* cell invasiveness, we perform Trans-well invasion assays for mir-182-5p antisense-transfected SF188 and KNS42 cells. The results reveal that mir-182 inhibition significantly downregulates the invasiveness of SF188 and KNS42 cells, by 58% and 51%, respectively (Fig. 3d).

## Discussion

Early detection and effective monitoring of pediatric gliomas may improve patient survival. Current diagnostic approaches often rely on invasive tissue biopsies and frequent neuroimaging, which may be harmful, particularly in children, thereby emphasizing the need for non-invasive alternatives. Liquid biopsy, based on the analysis of extracellular biomarkers in bodily fluids, offers a promising, less invasive alternative. Due to their differential expression, ability to cross an intact BBB, and stability in circulation, we considered circulating miRNAs as potential biomarkers for the detection of pediatric gliomas.

In this pilot study, we evaluated whether circulating miR-10b-5p, miR-182-5p, miR-21-5p, miR-106b-3p, and miR-25-3p are differentially expressed in pediatric glioma patients compared to HCs. These miRNAs have been reported to be upregulated in pHGG-4 tumors, and, excluding miR-21-5p, exhibit high circulating levels in adult GB patients. Our findings demonstrate for the first time that all five miRNAs are significantly upregulated in the plasma of pediatric glioma patients relative to HCs.

Expression analysis revealed differential patterns among glioma subtypes, with heterogeneous expression in the LGG patient group. Most LGG patients exhibited low plasma levels of all five miRNAs compared to pediatric HGGs. However, a subset of LGG patients displayed notably elevated miRNA expression levels. Further investigation suggests that this variability may correlate with distinct molecular subgroups within LGGs. Molecularly, most LGGs are characterized by BRAF translocations or fusions, and less commonly by BRAF activating mutations, typically presenting with low miRNA expression in our cohort. Conversely, other LGGs based on the tumor site or molecular characteristics formed distinct subgroups LGG.NF1, OG, BS&SC.LGG, and LGG.FGFR and exhibited varying expression profiles.

Notably, miR-182-5p and miR-25-3p levels were significantly elevated in HGGs and LGG.FGFRs, both of which are associated with poor prognoses. These results suggest a possible role for miR-182-5p and miR-25-3p in the aggressive behavior of these glioma subtypes. In addition, miR-106b-3p and miR-21-5p were predominantly upregulated in HGG patients, while miR-10b-5p was distinctively elevated in OG patients, suggesting subtype-specific associations.

ROC curve analyses indicated that all five circulating miRNAs could discriminate between HCs and pediatric glioma patients, with excellent discriminatory power for miR-182-5p, miR-25-3p, and miR-10b-5p, and good to very good discriminatory power for miR-106b-3p and miR-21-5p. However, when examining discrimination among glioma subtypes, variability was observed. Some subtypes exhibited only moderate diagnostic discrimination despite significant differential expression. This limitation may stem from the small sample sizes, amplifying the impact of individual variability and outliers. Expanding patient cohorts in future studies will be crucial to strengthen statistical power and confirm these preliminary findings.

By combining glioma subtypes that individually exhibited low diagnostic discrimination, we improved the overall discriminatory performance of the five circulating miRNAs. MiR-182-5p and miR-25-3p showed highly strong to excellent discrimination among HCs, LGGs, and other glioma subtypes, while miR-10b-5p excelled in distinguishing HCs from the total glioma group. Although miR-106b-3p and miR-21-5p demonstrated relatively lower discrimination ability, they nonetheless provided meaningful support in differentiating between certain pediatric groups.

**Fig. 4:**
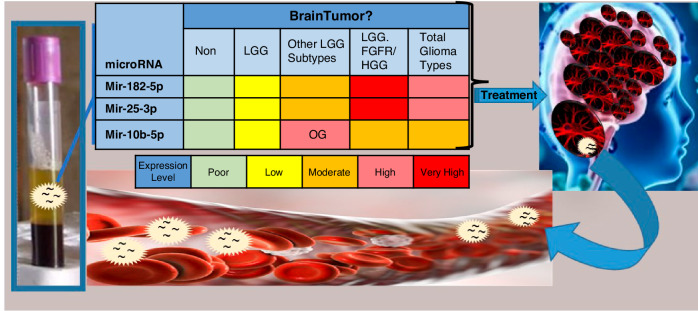
Schematic representation of a proposed diagnostic pipeline utilizing circulating microRNAs for pediatric glioma detection and classification. The figure illustrates a conceptual workflow wherein microRNAs are released from brain cells into the bloodstream. Blood samples are collected into standard tubes and processed for RNA extraction. The relative expression levels of five selected miRNA are measured and classified, according to cut off values, into five categories: very low, low, medium, high, or very high. The cut-off, envisioned by colors, is yet to be established through future large-cohort studies. Based on miRNA expression profiles, patients can be stratified into diagnostic categories, including no tumor, low-grade glioma (LGG), specific LGG subtypes (such as LGG with FGFR alterations), high-grade gliomas (HGG), or total gliomas for inclusion of all types. This future approach may assist in non-invasively diagnosing gliomas, monitoring tumor progression, evaluating treatment responses, detecting relapse, and guiding therapeutic decision-making, particularly in cases where brain biopsy is high-risk or infeasible.

These findings suggest that the five miRNAs could form the basis of a diagnostic algorithm for pediatric glioma detection and monitoring. Such a liquid biopsy test could provide clinicians with critical information in cases where a traditional biopsy is too risky, aiding in the differentiation of gliomas from benign lesions and in the distinction between LGGs and HGGs (Fig. 4).

Among the miRNAs studied, miR-182-5p emerged as the most promising biomarker, with significantly elevated plasma levels in glioma patients, particularly in HGGs, compared to HCs and LGGs. MiR-182-5p has previously been implicated in melanoma, medulloblastoma, osteosarcomas, ovarian, breast, pancreatic cancers, and adult gliomas, displaying a dual role as an oncogenic or suppressor in tumorigenesis.^39–41^

Here, we show that in pHGG-IV cells, miR-182-5p promotes migration and invasiveness, supporting its classification as an onco-miR in pediatric gliomas. Consequently, miR-182-5p inhibition emerges as a compelling therapeutic strategy. In future, miR-182-5p silencing may be achieved by antimiR oligonucleotides (AMOs), incorporating locked nucleic acids (LNAs) with phosphodiester-ribose backbone and 3’-end esterification. Additional approaches include miRNA masks that block target binding sites, and miRNA sponges or decoys that competitively inhibit mature miR-182-5p. Artificial miRNA (a-miRNA) sponges, designed with tandem repeats of miR-182-5p binding sites, can be expressed under the control of tissue-specific promoters, such as GFAP or Nestin, to restrict therapeutic activity to GB cells while minimizing systemic side effects. These innovative strategies warrant further investigation as potential treatments for pediatric GB or other CNS tumors.

Overall, our findings support the potential of circulating miRNAs as liquid biomarkers for pediatric glioma detection. This approach offers a valuable, less-invasive alternative to CSF ctDNA analysis, currently the most investigated method for CNS tumor diagnosis. In clinical scenarios where symptoms are subtle or nonspecific, circulating miRNA biomarkers could prompt earlier neuroimaging and diagnosis. While our pilot study demonstrates significant differences in plasma miRNA expression among glioma subtypes, the small sample sizes limit the robustness of the conclusions. Future larger-scale studies are essential to validate these findings across different clinical contexts, including pre- and post-surgery, during therapy, and at relapse.

## Supplementary information

Supplementary Information

## Data Availability

All data generated or analyzed during this study are included in this published article and its supplementary information files.
