# Supplementary material for: Circulating miRNAs as potential liquid biomarkers for pediatric gliomas
Source: Pediatr Res. 2025 Aug 20;99(3):1145–54. doi: 10.1038/s41390-025-04320-6 (PMC13021524; doi:10.1038/s41390-025-04320-6)
Supplement: Supplementary file 1 — Supplementary Information [file 41390_2025_4320_MOESM1_ESM.pdf]

Supplementary Table ST1: Summary of the case cohort demographic and clinical data

Glioma type/grade	Sex		Age (mean±SDEV)
	M	F	
Healthy control	7	5	12.5±6.1
Low-grade Glioma	31	20	10.33±6.61
High-grade Glioma	8	9	13.56±5.98

Supplementary Table ST2: Plasma expression averages, errors and ANOVA results for each circulating miRNA

(A)

mir-182-5p relative expression in pediatric gliomas							
	**HC**	**LGG**	**Optic Glioma**	**LGG. NF1**	**BS&SC. LGGs**	**LGG. FGFR**	**HGG**
**AV**	**0.468**	**1.552**	**5.349**	**7.367**	**5.889**	**16.512**	**16.0340**
**SD.ER**	0.301	0.194	1.549	2.603	1.475	7.022	4.5920

ANOVA summary mir-182-5p	
F	4.485
p value	0.0006
p value summary	***
Significant diff. among means (p<	Yes
R square	0.2694

Tukey's multiple comparisons test	Mean Diff.	95.00% CI of diff.	Adjusted *p-Value*
**HC vs. LGG.FGFR**	-16.04	-31.94 to -0.1497	0.0464
**HC vs. HGG**	-15.57	-27.55 to -3.581	0.0034
**LGG vs. HGG**	-14.48	-25.56 to -3.410	0.0031

(B)

mir-25-3p relative expression in pediatric gliomas							
	**HC**	**LGGs**	**Optic Glioma**	**LGG. NF1**	**BS&SC. LGGs**	**LGG. FGFR**	**HGGs**
**AV**	**0.889**	**3.320**	**8.532**	**6.979**	**10.643**	**15.927**	**17.049**
**SD.ER**	0.59	0.4700	1.6300	1.2100	2.3300	6.3200	3.3800

ANOVA summary mir-25-3p	
F	5.418
P value	0.0001
P value summary	***
Significant diff. among means (p< 0.05)?	Yes
R square	0.3171

Tukey's multiple comparisons test	Mean Diff.	95.00% CI of diff.	Adjusted *p-Value*
**HC** vs. **LGG.FGFR**	-13.09	-25.42 to -0.7649	0.0302
**HC** vs. **HGG**	-14.21	-23.91 to -4.515	0.0006
**LGG** vs. **LGG.FGFR**	-12.13	-23.93 to -0.3242	0.0401
**LGG** vs. **HGG**	-13.25	-22.27 to -4.226	0.0006

(C)

mir-10b-5p relative expression in pediatric gliomas							
	**HC**	**LGG**	**Optic Glioma**	**LGG. NF1**	**BS&SC. LGGs**	**LGG. FGFR**	**HGG**
**AV**	**0.526**	**2.127**	**7.076**	**2.356**	**5.288**	**3.558**	**3.789**
**SD.ER**	0.265	0.641	1.693	0.958	1.169	0.783	0.902

ANOVA summary mir-10b-5p	
F	4.295
p value	0.001
p value summary	**
Significant diff. among means (*p*< 0.05)?	Yes
R square	0.2808

Tukey's multiple comparisons test	Mean Diff.	95.00% CI of diff.	Adjusted *p-Value*
HC vs. Optic glioma	-6.55	-11.08 to -2.023	0.0008
HC vs. BS&SC.LGG	-4.763	-9.290 to -0.2360	0.0328
LGG vs. Optic glioma	-4.856	-8.968 to -0.7432	0.0107
LGG vs. HGG	-27.58	-50.71 to -4.442	0.0094
Optic glioma vs. HGG	-27.17	-52.80 to -1.541	0.0306
BS&SC.LGG vs. HGG	-25.64	-49.15 to -2.120	0.0237

(D)

mir-106b-3p relative expression in pediatric gliomas							
	HC	LGG	Optic Glioma	LGG. NF1	BS&SC. LGGs	LGG. FGFR	HGG
**AV**	**0.5602**	**0.924**	**1.331**	**2.783**	**2.917**	**6.69**	**28.5**
**SD.ER**	0.292	0.205	0.376	0.934	0.744	3.894	12.054

ANOVA summary mir-106b-3p	
F	3.291
p value	0.0062
p-value summary	**
Significant diff. among means (p < 0.05)?	Yes
R square	0.2063

Tukey's multiple comparisons test	Mean Diff.	95.00% CI of diff.	Adjusted *p-Value*
**HC vs. HGG**	-27.94	-53.65 to -2.234	0.0244
**LGG vs. HGG**	-27.58	-51.38 to -3.777	0.0129
**Optic glioma vs. HGG**	-27.17	-53.54 to -0.8040	0.0391

(E)

mir-21-5p relative expression in pediatric gliomas							
	HC	LGG	Optic Glioma	LGG .NF1	BS&SC. LGGs	LGG. FGFR	HGG
**AV**	**0.818**	**1.167**	**2.744**	**1.570**	**3.190**	**7.538**	**12.480**
**SD.ER**	0.236	0.158	0.6380	0.3130	0.7350	5.1500	5.7150

ANOVA summary mir-21-5p	
F	2.316
p value	0.0426
p value summary	*
Significant diff. among means (p< 0.05)?	Yes
R square	0.1656

Tukey's multiple comparisons test	Mean Diff.	95.00% CI of diff.	Adjusted *p-Value*
LGG vs. HGG	-11.31	-22.33 to -0.2995	0.0402

Supplementary Figure SF1: ROC curve analysis results summarized for each circulating miRNA

(A) mir-182-5p

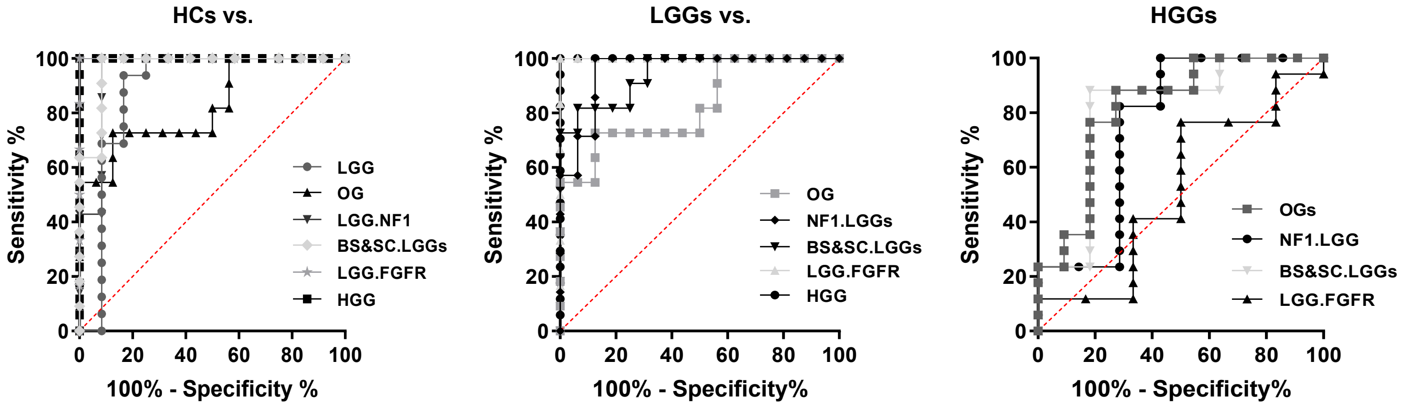

Area under the ROC curve -HC vs.	LGGs	OG	LGG.NF1	BS&SC.LGGs	LGG.FGFR	HGGs
Area	**0.885417**	**0.962121**	**0.952381**	**0.969697**	**1**	**1**
Std. Error	0.0825669	0.04019	0.0504408	0.0334926	0	0
95% confidence interval	0.724 to 1.000	0.883 to 1.000	0.8514 to 1.000	0.904 to 1.000	1.000 to 1.000	1.000 to 1.000
p value	0.0006	0.0002	0.0013	0.0001	0.0007	<0.0001
n=	16	11	7	11	6	17

Area under the ROC curve LGG (n=12) vs.	HC	OG	LGG.NF1	BS&SC.LGGs	LGG.FGFR	HGGs
Area	**0.8854**	0.8295	**0.955**	**0.943**	**1**	**1**
Std. Error	0.0826	0.0832	0.040	0.042	0.000	0.000
95% confidence interval	0.7236 to 1.000	0.666 to 0.993	0.878 to 1.000	0.8609 to 1.000	1.000 to 1.000	1.000 to 1.000
p value	0.0006	0.0042	0.0007	0.0001	0.0004	<0.0001
n=	12	11	7	11	6	16

Area under the ROC curve LGG vs.	HC	OG	LGG.NF1	BS&SC.LGGs	LGG.FGFR	HGGs
Area	**0.8854**	0.8295	**0.955**	**0.943**	**1**	**1**
Std. Error	0.0826	0.0832	0.040	0.042	0.000	0.000
95% confidence interval	0.7236 to 1.000	0.666 to 0.993	0.878 to 1.000	0.8609 to 1.000	1.000 to 1.000	1.000 to 1.000
p value	0.0006	0.0042	0.0007	0.0001	0.0004	<0.0001
n=	12	11	7	11	6	16

Area under the ROC curve HC (n=12) vs.	LGGs	OG +LGG.NF1 +BS&SC.LGG + LGG.FGFR +HGG
Area	**0.8854**	**0.9792**
Std. Error	0.0826	0.0211
95% confidence interval	0.7236 to 1.000	0.9378 to 1.000
p value	0.0006	<0.0001
n=	16	52

Area under the ROC curve LGG vs.	OG +LGG.NF1 +BS&SC.LGG +LGG.FGFR +HGG	BS&SC.LGGs + LGG.FGFR +HGG	LGG.FGFR +HGG
Area	**0.9459**	**0.9816**	**1**
Std. Error	0.0252795	0.01444	0.000
95% confidence interval	0.8964 to 0.9955	0.9533 to 1.000	1.000 to 1.000
p value	<0.0001	<0.0001	<0.0001
n=	52	34	23

Roc curve for mir-182-5p expression in LGG vs.	Cut-off [Rq]	Sensitivity %	95% CI	Specificity %	95% CI	Likelihood ratio
HC	> 0.5975	93.75	71.7% to 99.7%	83.3	55.2% to 97.%	5.63
OG +LGG.NF1+ LGG.FGFR +BS&SC.LGG + HGG	> 2.2445	90.4	79.4% to 95.82%	87.5	63.98% to 97.8%	7
BS&SC.LGGs + LGG.FGFR + HGG	> 2.894	94.12	80.9% to 98.95%	93.75	71.67% to 99.7%	15.06
LGG.FGFR + HGG	> 3.52050	100	85.7% to 100%	100	80.64% to 100%	

(B) mir-25-3p

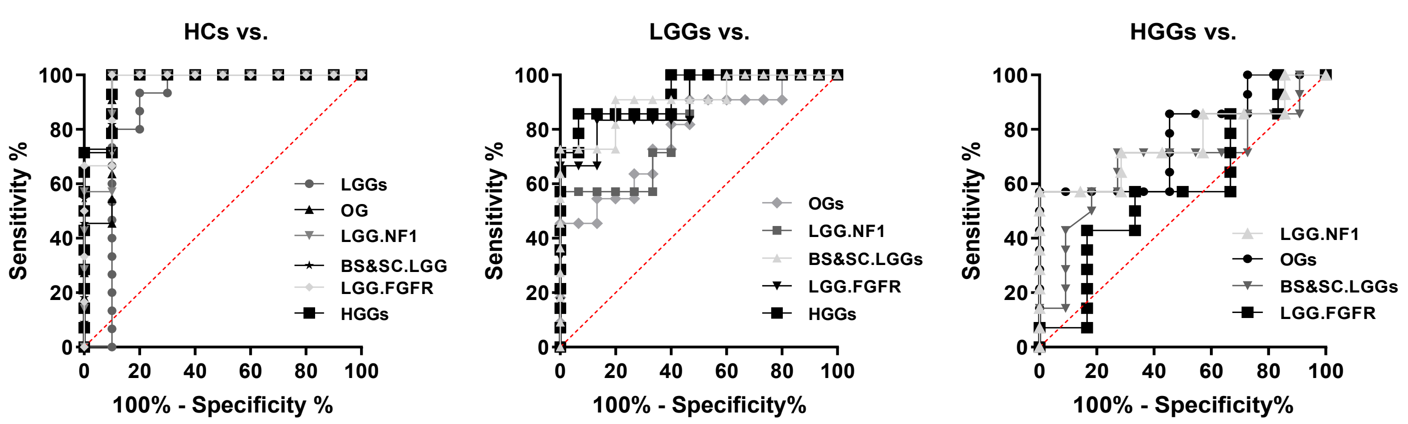

Area under the ROC curve HC vs.	LGG	Optic Glioma	LGG.NF1	LGG.FGFR	BS&SC.LGGs	HGGs
**Area**	**0.873**	**0.945**	**0.957**	**0.967**	**0.973**	**0.971**
**Std. Error**	0.096	0.056	0.048	0.041	0.032	0.032
**95% confidence interval**	0.686 to 1.000	0.8362 to 1.000	0.863 to 1.000	0.886 to 1.000	0.9104 to 1.000	0.909 to 1.000
p value	0.0019	0.0006	0.0018	0.0024	0.0003	0.0001
**n=**	15	11	6	6	11	14

Area under the ROC curve HC vs.	LGG	Optic Glioma	LGG.NF1	LGG.FGFR	BS&SC.LGGs	HGGs
**Area**	**0.873**	**0.945**	**0.957**	**0.967**	**0.973**	**0.971**
**Std. Error**	0.096	0.056	0.048	0.041	0.032	0.032
**95% confidence interval**	0.686 to 1.000	0.836 to 1.000	0.863 to 1.000	0.886 to 1.000	0.910 to 1.000	0.909 to 1.000
p value	0.0019	0.0006	0.0018	0.0024	0.0003	0.0001
**n=**	15	11	6	6	11	14

Area under the ROC curve HGGs vs.	HC	LGGs	OG	LGG .NF1	LGG. FGFR	BS&SC.LGG
**Area**	**0.971429**	**0.933333**	**0.766234**	0.7551	0.5	0.6786
**Std. Error**	0.031807	0.04527	0.096223	0.1053	0.1488	0.1111
**95% confidence interval**	0.909 to 1.000	0.845 to 1.000	0.578 to 0.955	0.549 to 0.961	0.208 to 0.792	0.461 to 0.896
p value	0.0001	<0.0001	0.0248	0.0622	>0.9999	0.1322
**n=**	10	15	11	7	6	11

Area under the ROC curve HC (n=10) vs.	LGG	OG +LGG.NF1 +BS&SC.LGG +LGG.FGFR +HGGs
**Area**	**0.873**	**0.963**
**Std. Error**	0.096	0.036
**95% confidence interval**	0.6855 to 1.000	0.893 to 1.000
p value	0.0019	<0.0001
**n=**	15	49

Area under the ROC curve LGG vs.	OG +LGG.NF1 +BS&SC.LGG +LGG.FGFR +HGGs	BS&SC. LGG +LGG.FGFR +HGG	LGG.FGFR +HGG
**Area**	**0.875**	**0.9183**	**0.9233**
**Std. Error**	0.045	0.0386	0.04296
**95% confidence interval**	0.788 to 0.962	0.843 to 0.994	0.839 to 1.000
p value	<0.0001	<0.0001	<0.0001
**n=**	49	31	20

Roc curve for mir-25-3p expression in LGGs vs.	Cut-off [Rq]	Sensitivity %	95% CI	Specificity %	95% CI	Likelihood Ratio
**HC**	> 1.515	95.3	87.1% to 98.7%	90	59.6% to 99.5%	9.5
**OG +LGG.NF1 +LGG.FGFR +BS&SC.LGG +HGGs**	> 4.285	81.6	68.6% to 90.%	66.7	41.7% to 84.8%	2.4
**BS&SC.LGG + LGG.FGFR +HGGs**	> 4.890	87.1	71.2% to 94.9%	80	54.8% to 92.95%	4.4
**LGG.FGFR +HGGs**	> 5.520	85	63.96% to 94.8%	86.7	62.% to 97.6%	6.4
**HGGs**	> 5.947	85.7	60.% to 97.5%	93.3	70.2% to 99.7%	12.9

(C) mir-10b-5p

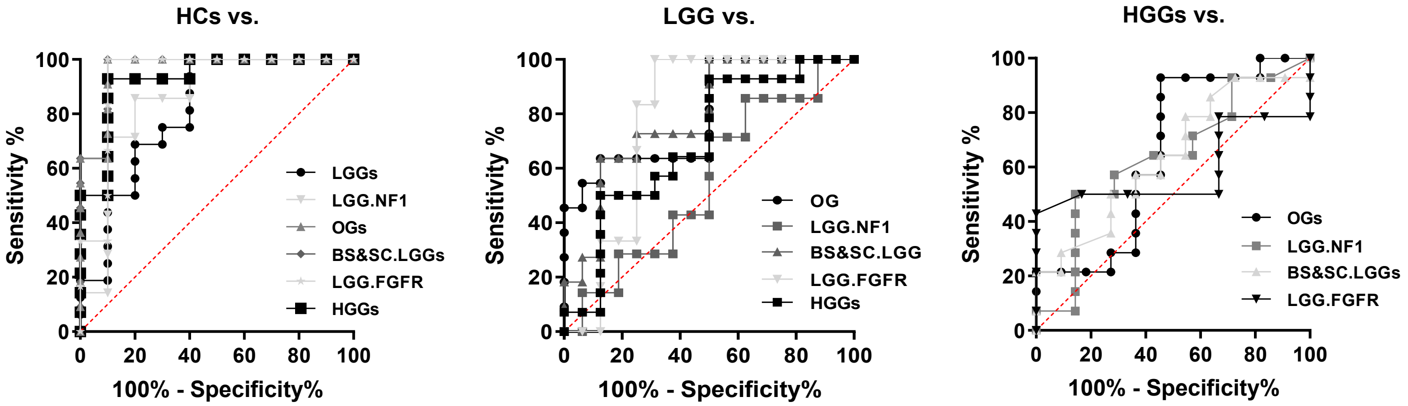

**Area under the ROC curve HC vs.**	**LGG**	**Optic glioma**	**LGG.NF1**	**LGG.FGFR**	**BS&SC. LGG**	**HGG**
**Area**	**0.8125**	**0.963636**	0.857143	**0.933333**	**0.963636**	**0.928571**
**Std. Error**	0.0942176	0.0398721	0.0955573	0.0685836	0.0398721	0.0560872
**95% confidence interval**	0.6278 to 0.997	0.886 to 1.000	0.6699 to 1.000	0.799 to 1.000	0.886 to 1.000	0.819 to 1.000
p value	0.0084	0.0003	0.0147	0.0048	0.0003	0.0004
**n=**	16	11	7	6	11	14

**Area under the ROC curve - LGG vs.**	**HC**	**Optic glioma**	**LGG.NF1**	**LGG.FGFR**	**BS&SC.LGG**	**HGG**
**Area**	**0.813**	0.801	0.554	0.781	**0.790**	**0.888**
**Std. Error**	0.094	0.088	0.128	0.098	0.088	0.060
**95% confidence interval**	0.628 to 0.997	0.628 to 0.974	0.303 to 0.8043	0.5898 to 0.973	0.618 to 0.962	0.7701 to 1.000
p value	0.0084	0.0089	0.6885	0.0465	0.0118	0.0003
**n=**	10	11	7	6	11	14

**Area under the ROC curve HGG vs.**	**HC**	**LGG**	**Optic glioma**	**LGG.NF1**	**LGG.FGFR**	**BS&SC. LGG**
**Area**	**0.9286**	0.6964	0.6558	0.638	0.5893	0.6396
**Std. Error**	0.05609	0.09805	0.11902	0.1327	0.1273	0.1126
**95% confidence interval**	0.819 to 1.000	0.5043 to 0.889	0.423 to 0.889	0.378 to 0.898	0.3397 to 0.839	0.419 to 0.8603
p value	0.0004	0.0674	0.1889	0.3139	0.5362	0.2392
**n=**	10	16	11	7	6	11

D. mir-106b-3p

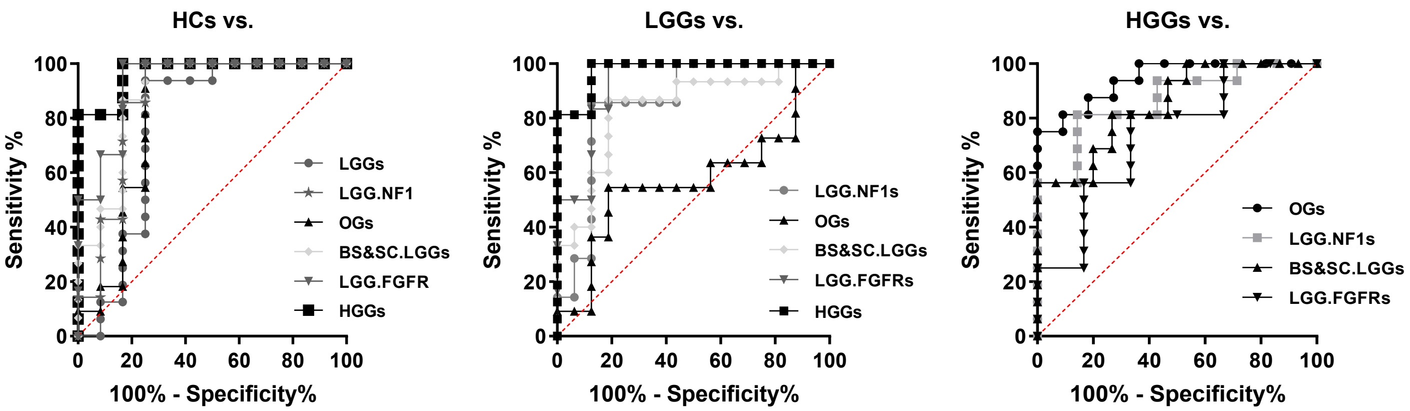

Area under the ROC curve HCs vs.	LGGs	OG	LGG.NF1	BS&SC.LGG	LGG.FGFR	HGGs
Area	0.776042	0.818182	**0.869048**	**0.888889**	**0.930556**	**0.96875**
Std. Error	0.107631	0.0993858	0.0855018	0.0701312	0.0592204	0.0279569
95% confidence interval	0.5651 to 0.987	0.6234 to 1.000	0.7015 to 1.000	0.7514 to 1.000	0.815 to 1.000	0.914 to 1.000
p value	0.0139	0.0097	0.0088	0.0006	0.0037	<0.0001
n=	12	11	7	15	6	12

Area under the ROC curve LGGs vs.	HC	OG	LGG .NF1	BS&SC.LGG	LGG.FGFR	HGG
Area	0.776042	0.573864	0.857143	0.8375	0.927083	0.976563
Std. Error	0.107631	0.121473	0.08191	0.0747977	0.0551711	0.0213117
95% confidence interval	0.565 to 0.987	0.3358 to 0.812	0.697 to 1.000	0.691 to 0.984	0.8195 to 1.000	0.935 to 1.000
p value	0.0139	0.5212	0.0075	0.0014	0.0025	<0.0001
n=	12	11	7	15	6	16

Area under the ROC curve HGG vs.	HC	LGGs	OG	LGG .NF1	LGG. FGFR	BS& SC. LGG
Area	**0.96875**	**0.976563**	**0.943182**	**0.866071**	0.739583	**0.85**
Std. Error	0.0279569	0.0213117	0.0404268	0.0777231	0.124824	0.0671036
95% confidence interval	0.914 to 1.000	0.935 to 1.000	0.864 to 1.000	0.714 to 1.000	0.495 to 0.984	0.7185 to 0.982
P value	<0.0001	<0.0001	0.0001	0.0062	**0.09**	0.0009
n=	12	16	11	7	6	15

Area under the ROC curve HC +LGG +OG +NF1 + BS&SC.LGG (n=67)	HGG + LGG.FGFR
Area	0.8947
Std. Error	0.03643
95% confidence interval	0.8233 to 0.9661
P value	<0.0001
n=	21

Roc curve for mir-106b-3p expression in HGG + LGG.FGFR	Cut-off [Rq]	Sensitivity %	95% CI	Specificity %	95% CI	Likelihood ratio
HC +LGG +OG +NF1 + BS&SC.LGG	> 1.999	85.7	65.4% to 95.%	68.42	57.3% to 77.8%	2.7

(E) mir-21-5p

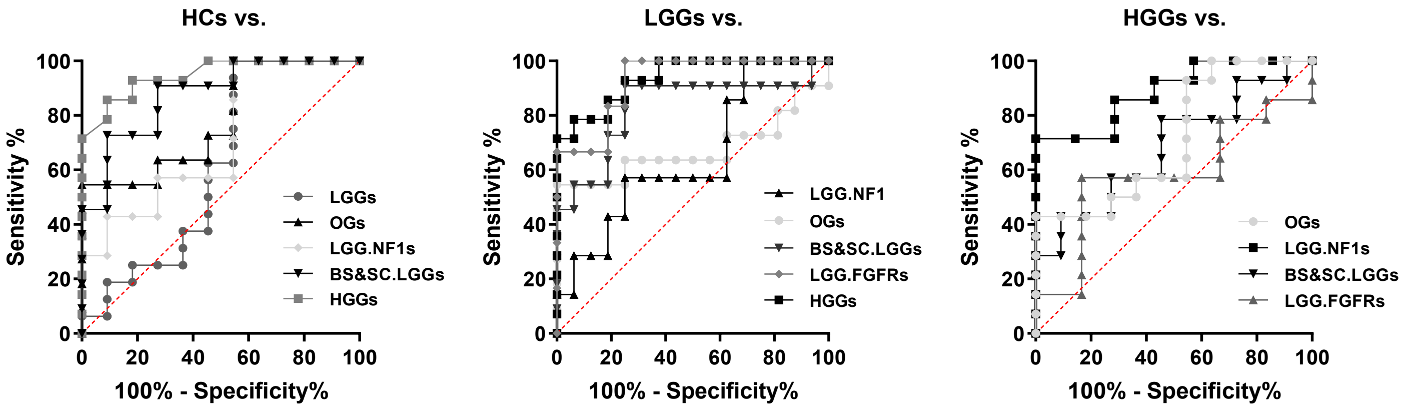

Area under the ROC curve HC vs.	LGG	Optic Glioma	LGG.NF1	BS&SC.LGG	LGG.FGFR	HGGs
Area	0.613636	**0.785124**	0.714286	**0.876033**	**0.954545**	**0.9048**
Std. Error	0.12365	0.0989057	0.126192	0.0737284	0.0467745	0.0595025
95% confidence interval	0.3713 to 0.856	0.591 to 0.979	0.467 to 0.962	0.7315 to 1.000	0.863 to 1.000	0.788 to 1.000
p value	0.3237	**0.0235**	0.1351	**0.0028**	**0.0026**	**0.0005**
n=	16	11	7	11	6	14

Area under the ROC curve LGG vs.	HC	Optic Glioma	BS&SC.LGG	LGG.FGFR	HGGs
Area	0.5625	0.676136	**0.829545**	**0.927083**	**0.9375**
Std. Error	0.123522	0.123983	0.0902245	0.057577	0.0408898
95% confidence interval	0.3204 to 0.805	0.433 to 0.919	0.653 to 1.000	0.8142 to 1.000	0.8574 to 1.000
p value	0.5775	0.1261	0.0042	0.0025	<0.0001
n=	11	11	11	6	14

Area under the ROC curve HGGs vs.	HC	LGG	Optic Glioma	LGG.NF1	BS&SC. LGG	LGG.FGFR
Area	**0.948052**	**0.9375**	0.714286	**0.887755**	0.681818	0.583333
Std. Error	0.0403918	0.0408898	0.10566	0.0713914	0.107478	0.135642
95% confidence interval	0.869 to 1.000	0.857 to 1.000	0.5072 to 0.9212	0.748 to 1.000	0.4712 to 0.893	0.3175 to 0.849
p value	0.0002	<0.0001	0.0708	0.0046	0.1253	0.5637
n=	11	16	11	7	11	6

Area under the ROC curve HC +LGG +LGG.NF1 vs.	OG +BS&SC.LGG LGG.FGFR +HGGs	BS&SC.LGG + LGG.FGFR +HGG	LGG.FGFR +HGGs
Area	**0.843137**	**0.877419**	**0.7922**
Std. Error	0.0448906	0.0423632	0.05614
95% confidence interval	0.7552 to 0.931	0.7944 to 0.9605	0.6822 to 0.9023
p value	<0.0001	<0.0001	0.0002
n=	42	31	20

Roc curve for mir-21-5p expression in HC +LGGs +LGG.NF1 vs.	Cut-of [Rq]	Sensitivity %	95% CI	Specificity %	95% CI	Likelihood ratio
OG +BS&SC.LGG LGG.FGFR +HGGs	> 1.2010	85.7	72.2% to 93.3%	67.7	50.8% to 80.9%	2.65
BS&SC.LGG + LGG.FGFR +HGG	> 1.2800	93.5	79.3% to 98.85%	68.6	52% to 81.45%	2.98
LGG.FGFR + HGGs	> 1.4255	95	76.4% to 99.7%	71.4	54.9% to 83.7%	3.3

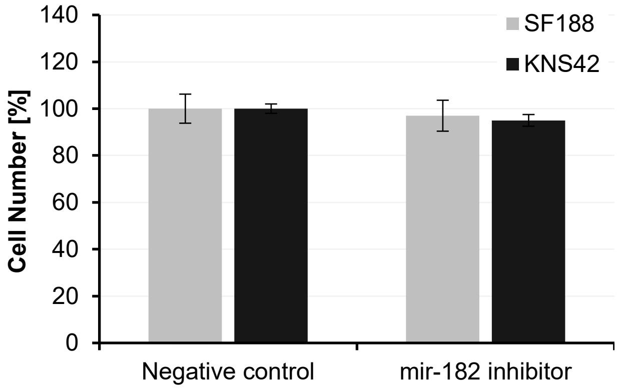

Supplementary Figure SF2: ***pHGG-IV cell viability following treatment with candidate miRNA.*** KNS42 and SF188 cell lines were transfected with hsa-miR-182-5p antisense LNA oligonucleotide or scrambled LNA oligonucleotide (Negative control A) as negative control (50nM). After 72h of incubation the cells were harvested and each received cell sample was counted using trypan blue exclusion assay: 20µl cell suspension (in medium) were diluted with 20µ trypan blue (1:1 dilution), and 10µl of the mixed suspension were inserted to hemocytometer, for cell counting under microscope).
